# Case Report: Physiological and psychological underpinnings of muscle dysmorphia using EEG, GSR, and eye-tracking

**DOI:** 10.3389/fpsyg.2025.1553997

**Published:** 2025-07-21

**Authors:** Metin Çınaroğlu, Selami Varol Ülker, Eda Yılmazer, Gökben Hızlı Sayar

**Affiliations:** ^1^Psychology Department, İstanbul Nişantaşı University, İstanbul, Türkiye; ^2^Neuro Marketing Research Laboratory, Psychology Department, Üsküdar University, İstanbul, Türkiye; ^3^Psychology Department, Beykoz University, İstanbul, Türkiye; ^4^Medical School, Üsküdar University, İstanbul, Türkiye

**Keywords:** case report, muscle dysmorphia, body dysmorphic disorder, electroencephalography, galvanic skin response, eye-tracking

## Abstract

**Background:**

Muscle dysmorphia (MD), a subtype of Body Dysmorphic Disorder (BDD), involves an obsessive preoccupation with perceived insufficient muscularity despite an objectively muscular physique. While its psychological features are well-documented, physiological and attentional underpinnings remain underexplored.

**Objective:**

This exploratory, proof-of-concept case series examines the psychological, physiological, and attentional characteristics of individuals with varying experiences of MD using a multimodal approach combining electroencephalography (EEG), galvanic skin response (GSR), and eye-tracking technologies.

**Methods:**

Three male participants were purposefully selected to represent distinct clinical profiles: one with active MD and steroid use, one in sustained remission from MD, and one with no MD history. Participants completed validated psychological scales (MDDI, BIDQ, STAI, RSES) and were exposed to personalized visual stimuli (past, current, and idealized body images). A triangulated recording protocol was used to capture EEG, GSR, and eye-tracking data during stimulus exposure.

**Results:**

Participants with current and past MD showed elevated beta wave activity, increased skin conductance, and attentional biases toward muscular regions, corresponding with higher self-reported distress and anxiety. In contrast, the control participant exhibited stable physiological responses and emotionally neutral reactions. Triangulated data revealed coherent patterns across subjective and physiological domains, supporting the internal validity of the findings despite the small sample.

**Conclusion:**

These findings illustrate the potential of multimodal assessment in identifying candidate psychophysiological markers of MD. While not generalizable, this case-series provides a valuable framework for future hypothesis-driven research and supports the need for gender-specific diagnostic and intervention strategies in muscle dysmorphia.

## 1 Introduction

MD, a subtype of BDD, is characterized by an obsessive preoccupation with insufficient muscularity, despite often possessing a visibly muscular physique (Sreshta et al., 2017). Initially considered a male parallel to eating disorders (Murray et al., 2010), MD is now recognized as a complex condition impacting cognitive, emotional, and behavioral domains (Rück et al., 2024). Individuals with MD often engage in compulsive exercise (Martenstyn et al., 2022a), adhere to rigid dietary regimens (Martenstyn et al., 2022b), and may resort to anabolic steroid use (Zaiser et al., 2024), resulting in significant psychological distress (Cuadrado et al., 2024) and physical health risks (Bonnecaze et al., 2021).

While extensive research has explored the psychological dimensions of MD (Prnjak et al., 2022), including self-esteem issues (Greenway and Price, 2020), anxiety (Zheng et al., 2021), and body image dissatisfaction (Dal Brun et al., 2024), the physiological and neurocognitive underpinnings of MD remain poorly understood. To the best of our knowledge no studies have examined brain activity, autonomic arousal, and visual attention patterns in individuals with MD, leaving a gap in understanding the interaction between psychological symptoms and physiological responses.

This study is framed as an exploratory, proof-of-concept investigation aimed at generating hypotheses about the physiological markers of MD using a multimodal design. Given the rarity and complexity of muscle dysmorphia, especially among individuals actively using anabolic steroids, a case study design was deemed appropriate for this exploratory investigation. The use of a small, strategically selected sample allowed for an in-depth, multimodal examination of distinct MD presentations—active, remitted, and asymptomatic. This methodological choice is consistent with the goals of early-stage clinical neuroscience research, where detailed physiological and psychological profiling of hard-to-reach populations serves as a foundation for hypothesis generation and future large-scale studies. Importantly, this design provides contextual nuance and ecological validity, capturing the lived experiences of individuals whose psychological symptoms are often concealed or normalized in performance-oriented subcultures. While the findings are not intended for generalization, they offer critical insights into the neurophysiological correlates of MD, which remain virtually undocumented in the existing literature.

This case report employs a multimodal assessment approach, integrating EEG, GSR, and eye-tracking technology, combined with validated psychological scales MDDI (Devrim and Bilgic, 2019), BIDQ (Kuzu et al., 2020), STAI (Döner et al., 2024), RSES (Gnambs et al., 2018). Three participants, representing varying experiences with MD—an active MD sufferer with steroid use, a former MD sufferer post-recovery, and a control participant with no MD symptoms—were exposed to personalized visual stimuli of their past, current, and idealized body images.

By analyzing both self-reported psychological data and objective physiological markers, this study aims to identify patterns of emotional and cognitive responses associated with MD, shedding light on the neurocognitive and physiological mechanisms underlying body image distress. The findings aim to contribute to the development of targeted interventions and early detection strategies for individuals affected by MD.

### 1.1 Case descriptions

#### 1.1.1 Case 1: E.S.—active MD diagnosis

E.S., a 24-year-old male, has been actively bodybuilding for 6 years and using anabolic steroids and performance-enhancing drugs (PEDs) since age 20. He follows a rigid six-day training regimen and a strict diet. Despite his objectively muscular physique, E.S. reports persistent body dissatisfaction, obsessive self-scrutiny, and social withdrawal driven by perceived inadequacies in his physical appearance. Psychological assessments revealed severe symptoms of muscle dysmorphia, significant body image distress, elevated anxiety levels, and low self-esteem. E.S. described intense emotional responses during visual exposure to body images, including regret, frustration, and a strong reliance on steroids despite awareness of their adverse effects.

#### 1.1.2 Case 2: A.K.—post-MD recovery

A.K., a 44-year-old male, has a history of competitive bodybuilding and anabolic steroid use but discontinued both over 6 years ago. He now leads a sedentary lifestyle and reflects on his past with a mix of pride and regret. In this study, “recovery” was operationally defined as the cessation of pathological behaviors associated with muscle dysmorphia (e.g., steroid use, compulsive training), return to a non-body-centric lifestyle, and scoring below clinical thresholds on validated psychological measures for MD, sustained for at least 12 months. A.K. met these criteria, though he continues to report occasional intrusive thoughts and mild emotional discomfort related to body image. Psychological assessments indicated moderate residual symptoms of muscle dysmorphia, moderate anxiety, and fluctuating self-esteem. His emotional reflections revealed some lingering cognitive patterns—such as frustration over physical changes and intermittent preoccupation with his former physique—that do not currently interfere with daily functioning or meet diagnostic criteria.

#### 1.1.3 Case 3: M.T.—neutral and no MD diagnosis

M.T., a 39-year-old male, engages in recreational fitness three times a week with no history of steroid use or body dysmorphic concerns. He describes his relationship with fitness as health-oriented and non-obsessive. Psychological assessments revealed no signs of muscle dysmorphia, low anxiety levels, and high self-esteem. M.T. reported neutral emotional responses during visual exposure to body images, emphasizing contentment with his physical appearance and an absence of distress or fixation on specific body features.

## 2 Materials and methods

### 2.1 Participants

Three male participants were purposefully selected to represent distinct profiles relevant to MD, BDD, and fitness behaviors. The inclusion of only male participants was intentional, as muscle dysmorphia is predominantly observed in men, and the study aimed to minimize gender-related variability in this exploratory case-series design. Recruitment occurred via referrals from fitness professionals, licensed clinicians, and targeted outreach at fitness facilities. Eligibility was assessed through an online screening questionnaire evaluating fitness habits, steroid use, body image concerns, and psychological well-being, followed by a structured clinical interview conducted by one of the authors (GHS), a licensed psychiatrist, using DSM-5 diagnostic criteria for Body Dysmorphic Disorder, with specific attention to the muscle dysmorphia subtype. Although the SCID-5 was not formally administered, the interview systematically assessed the key DSM-5 criteria for BDD: (1) preoccupation with perceived appearance flaws; (2) engagement in repetitive behaviors or mental acts; (3) clinically significant distress or impairment; and (4) in the case of muscle dysmorphia, a focus on perceived insufficient muscularity. The final sample included a 24-year-old with active MD, ongoing steroid use, and a strict six-day training and diet regimen; a 44-year-old with a past MD diagnosis who discontinued steroid use and bodybuilding 6 years ago and now leads a sedentary lifestyle; and a 39-year-old recreational gym-goer with no history of MD, steroid use, or body image concerns. Participants met the inclusion criteria of being male, aged 18–65, capable of informed consent, and free from psychiatric medication or ongoing psychotherapy. Exclusion criteria included neurological disorders, severe medical conditions, or an inability to comply with the study protocol.

### 2.2 Study design and procedure

The study employed a case study design, adhering to CARE guidelines for methodological rigor (Riley et al., 2017). It comprised three phases: baseline psychological assessment, experimental image-viewing task, and post-exposure emotional assessment. In the baseline phase, participants completed validated self-report instruments (MDDI, BIDQ, STAI, RSES) to evaluate symptoms of muscle dysmorphia, body image distress, anxiety, and self-esteem. This phase was followed by a resting-state recording period, during which EEG, GSR, and eye-tracking data were collected under neutral conditions (e.g., blank screen, eyes open/closed, static neutral stimulus) to establish individualized baseline physiological profiles prior to stimulus exposure. During the experimental phase, participants viewed three personalized body images—past, current, and idealized—each displayed for 30 s on a calibrated high-resolution monitor, interleaved with 10-s neutral gray screens to minimize emotional carryover. EEG, GSR, and eye-tracking metrics were recorded continuously throughout the task to capture real-time physiological and attentional responses. In the post-exposure phase, participants rated their emotional reactions using a Visual Analog Scale (VAS) and took part in semi-structured interviews to qualitatively explore their cognitive and emotional responses during image viewing. To enhance methodological rigor and internal validity, the study implemented a triangulation framework that integrated subjective psychological assessments with neurophysiological (EEG), autonomic (GSR), and attentional (eye-tracking) data sources. This multimodal approach allowed for the identification of converging patterns across distinct response systems and supported the interpretive robustness of findings, despite the study’s limited sample size.

### 2.3 Experimental setup and procedure

The experimental phase took place at the Üsküdar University Psycho-Physical Research Laboratory, utilizing EEG, GSR, and eye-tracking technologies, integrated through the iMOTIONS 10.0.1. Participants were seated in a soundproof, controlled environment, approximately 60 cm from a calibrated high-resolution monitor, ensuring session consistency. Participants viewed three personalized images—past, current, and idealized body images—in random order. Each image was displayed for 30 s, separated by a 10-s neutral gray screen to minimize emotional carryover. The trial structure included one exposure per image condition (past, current, idealized) for each participant. This single-trial design was chosen based on the personalized and emotionally evocative nature of the stimuli, which were derived from participants’ own body history and ideals. In line with ethical standards and similar research in clinical populations, repeated exposures were avoided to prevent emotional habituation, fatigue, or distress—particularly for individuals with active or residual MD symptoms. Prior literature on body image and affective neuroscience confirms that single, high-salience trials are sufficient to elicit meaningful physiological responses when data are recorded continuously and analyzed relative to individual baselines. This design preserved ecological validity while minimizing potential carryover effects, allowing for clear within-subject comparisons across the three image types.

Participants were instructed to remain still and focused throughout the task. Physiological data were continuously recorded: EEG captured beta wave activity (13–30 Hz) in frontal and parietal regions for cognitive and emotional processing, as altered beta wave patterns have been linked to heightened anxiety (Leaf et al., 2023) and obsessive thought processes (Perera et al., 2023); GSR measured skin conductance changes reflecting emotional arousal, which indicates physiological responses to emotional stimuli (Shehu et al., 2023); and eye-tracking analyzed fixation durations and gaze patterns across predefined areas of interest (e.g., chest, arms, abdomen), highlighting areas of attentional bias associated with body dissatisfaction (Wong et al., 2022). After each image, participants rated their emotional distress on a (0–10) VAS (Lukacz et al., 2004) and participated in a semi-structured interview (Magaldi and Berler, 2020) to share reflections on their emotional and cognitive experiences.

### 2.4 Laboratory equipment

The study employed the Emotiv Epoc X EEG headset, Shimmer 3 GSR + Unit, and iMOTIONS 10.0.1 platform for synchronized data acquisition and analysis. The Emotiv Epoc X recorded EEG signals from 14 channels with two reference sensors (CMS/DRL at P3/P4). It operated with a 14-bit resolution (0.51 μV) or an optional 16-bit resolution (0.1275 μV), covering a frequency range of 0.16–43 Hz. The Shimmer 3 GSR + Unit measured skin conductance using two Ag/AgCl electrodes attached to the non-dominant hand’s middle and index fingers. It operated within a resistance range of 10kΩ–4.7 MΩ (100uS–0.2uS conductance) and featured Bluetooth connectivity and onboard data storage. Eye-tracking technology analyzed fixation durations, gaze patterns, and heatmaps on predefined areas of interest (chest, arms, abdomen). The soundproof and temperature-controlled laboratory ensured consistent lighting. Participants sat 60 cm from a calibrated monitor, with all equipment calibrated before each session for data reliability.

### 2.5 Data analysis

EEG data were pre-processed to remove artifacts and filtered (0.5–50 Hz) for spectral analysis, focusing on beta wave power across targeted regions. Baseline EEG, GSR, and eye-tracking data were analyzed to confirm stable physiological states prior to image exposure, serving as individualized reference points. To address potential sensor noise and reduce the impact of inter-individual physiological variability, all outcome measures were computed as changes from baseline within each participant. This approach ensured that comparisons reflected stimulus-specific reactivity rather than raw signal values, which may vary across individuals due to anatomical, behavioral, or device-related factors. GSR data were analyzed for peak amplitudes using the transformation SCR = log(1 + |SC|) and for response latencies during stimulus presentation. Eye-tracking data were processed to extract fixation durations and generate gaze heatmaps across predefined areas of interest (e.g., chest, arms, abdomen). Repeated-measures ANOVA with post-hoc pairwise tests was conducted to compare responses across the three image types. Mixed-effects models were used to account for variability in eye-tracking data, and Pearson’s correlation coefficients were calculated to assess associations between physiological markers and psychological scale scores. Psychological scale results were summarized descriptively with means and standard deviations and interpreted in conjunction with physiological findings.

### 2.6 Ethical considerations

The study was approved by the Institutional Review Board of Üsküdar University (No: 61351342/020-318). Written informed consent was obtained from all participants, who were informed of their right to withdraw at any time without consequence. A debriefing session followed the experimental phase to address any distress, with psychological support offered if needed. Data were anonymized, securely stored, and managed according to the Declaration of Helsinki and CARE guidelines.

## 3 Results

**Figure 1:**
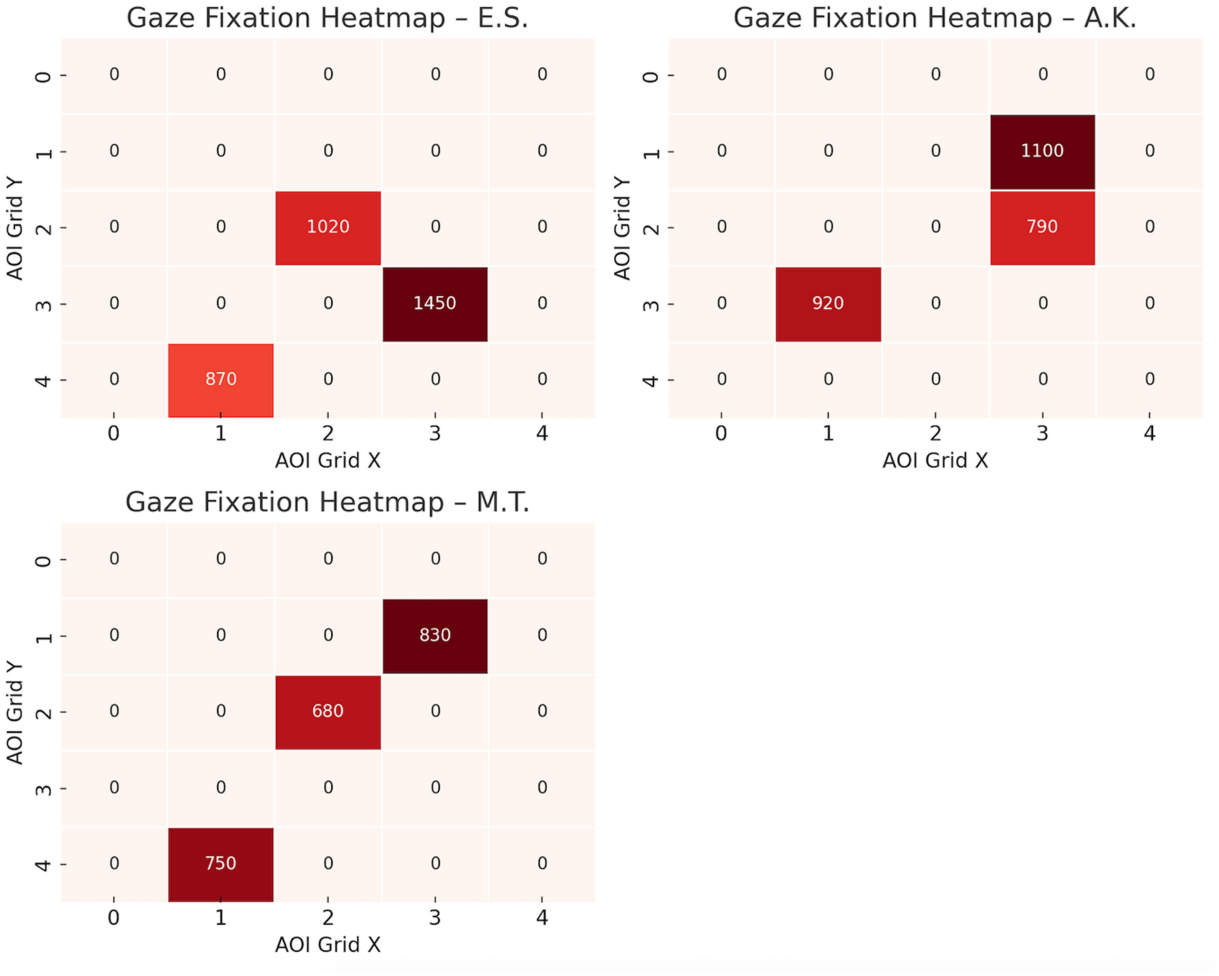
Gaze fixation heatmaps.

Baseline measures showed stable beta wave activity across frontal and parietal regions, low and consistent GSR levels indicating minimal emotional arousal, and evenly distributed eye-tracking fixation patterns. These baselines confirmed neutral physiological states before stimulus presentation (Figure 1).

The gaze fixation heatmaps visualize cumulative attention distribution across predefined body regions using a 5 × 5 AOI grid. Case E.S. (active MD) showed highly concentrated visual attention on upper-body regions, with particularly intense focus on chest and arm areas. Case A.K. (post-recovery) exhibited a more distributed but still upper-body-biased fixation pattern. In contrast, Case M.T. (control) displayed relatively balanced attention, with less pronounced clustering and lower overall fixation density. These heatmaps reflect participant-specific attentional biases that align with their clinical status and complement the physiological data reported.

### 3.1 Case 1: E.S.

Baseline EEG data revealed moderate beta wave activity across frontal (10.2 μV^2^, SD = 2.5), parietal (9.6 μV^2^, SD = 2.1), and occipital (8.8 μV^2^, SD = 2.0) regions during the neutral state. During the image-viewing task, beta wave activity significantly increased, with the highest activity observed during the idealized image (Frontal: 15.6 μV^2^, SD = 4.1; Parietal: 13.8 μV^2^, SD = 3.1; Occipital: 18.2 μV^2^, SD = 3.7; *p* < 0.01). GSR baseline levels indicated minimal arousal (SCR Amplitude: 0.9 μS, SD = 0.3), while exposure to body images led to significantly elevated SCR peaks, peaking during the idealized image (3.1 μS, SD = 0.8, *p* < 0.01). Eye-tracking baseline revealed evenly distributed gaze patterns with brief fixations (Mean Fixation Duration: 420 ms, Number of Fixations: 8). During image exposure, prolonged fixations were observed on chest and arm regions, particularly during the idealized image (Fixation Duration: 1,450 ms, Number of Fixations: 24).

Psychological assessments showed severe symptoms of muscle dysmorphia (MDDI: 51/60), significant body image distress (BIDQ: 6.2/7), high anxiety levels (STAI-State: 52/80; STAI-Trait: 58/80), and very low self-esteem (RSES: 10/30). Correlation analysis revealed strong positive associations between MDDI scores and frontal beta activity (*r* = 0.72, *p* < 0.01) and BIDQ scores with SCR peaks (*r* = 0.65, *p* < 0.01).

VAS scores indicated severe emotional distress across images, with the highest reported distress for the idealized image (10/10). Semi-structured interviews revealed obsessive preoccupations with muscular inadequacy, reliance on steroids, and heightened anxiety linked to perceived flaws.

**Figure 2:**
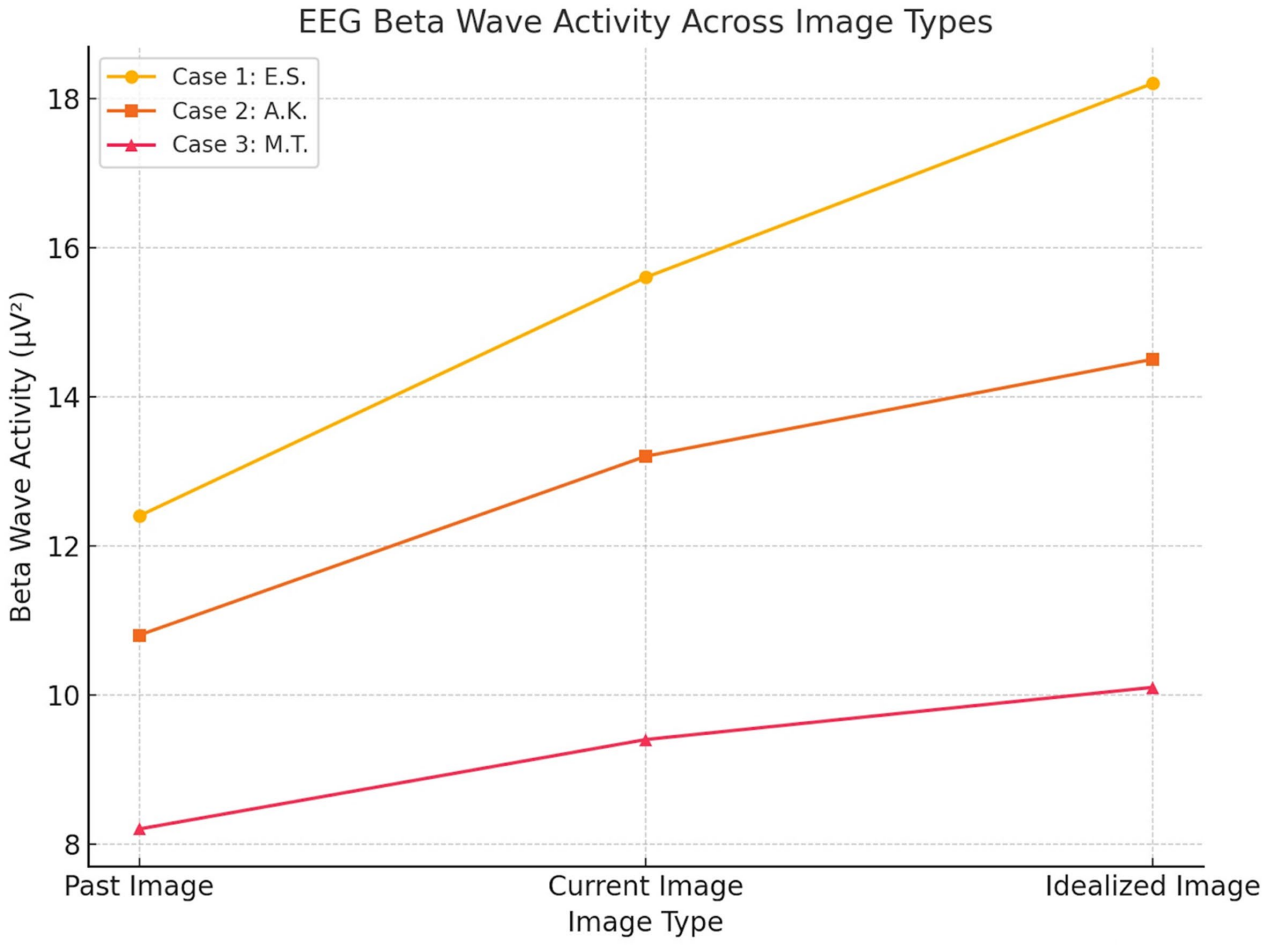
EEG beta wave activity.

EEG beta wave activity (13–30 Hz) across image types (Past, Current, Idealized) for each participant. Case 1 (E.S., active MD) exhibited the highest beta activity, particularly in response to the idealized body image, while Case 2 (A.K., recovered MD) showed moderate elevation. Case 3 (M.T., control) demonstrated stable, low beta activity across conditions (Figure 2).

### 3.2 Case 2: A.K.

Baseline EEG data revealed low-to-moderate beta wave activity across frontal (9.1 μV^2^, SD = 2.2), parietal (8.7 μV^2^, SD = 2.0), and occipital (8.9 μV^2^, SD = 2.1) regions during the neutral state. During the image-viewing task, beta wave activity increased moderately, peaking during the idealized image (Frontal: 13.2 μV^2^, SD = 3.1; Parietal: 12.5 μV^2^, SD = 2.8; Occipital: 14.5 μV^2^, SD = 3.4; *p* < 0.05). Baseline GSR responses showed low arousal levels (SCR Amplitude: 0.7 μS, SD = 0.2), with significant increases observed during the current image (2.1 μS, SD = 0.5; *p* < 0.05). Eye-tracking baseline indicated balanced gaze distribution and brief fixations (Mean Fixation Duration: 390 ms, Number of Fixations: 7). During image exposure, fixations became more focused on perceived weaker areas such as the arms and abdomen, particularly during the current image (Fixation Duration: 920 ms, Number of Fixations: 16).

Psychological assessments reflected mild symptoms of muscle dysmorphia (MDDI: 18/60), moderate body image distress (BIDQ: 3.8/7), moderate state anxiety (STAI-State: 40/80), and moderate trait anxiety (STAI-Trait: 43/80), accompanied by low-average self-esteem (RSES: 16/30). Correlation analysis indicated a moderate association between BIDQ scores and SCR amplitudes (*r* = 0.54, *p* < 0.05).

VAS scores indicated moderate emotional distress, ranging from 5/10 for the past image to 7/10 for the current image. Semi-structured interviews revealed lingering emotional vulnerability, occasional distress related to physical changes, and a sense of loss tied to past physique ideals.

**Figure 3:**
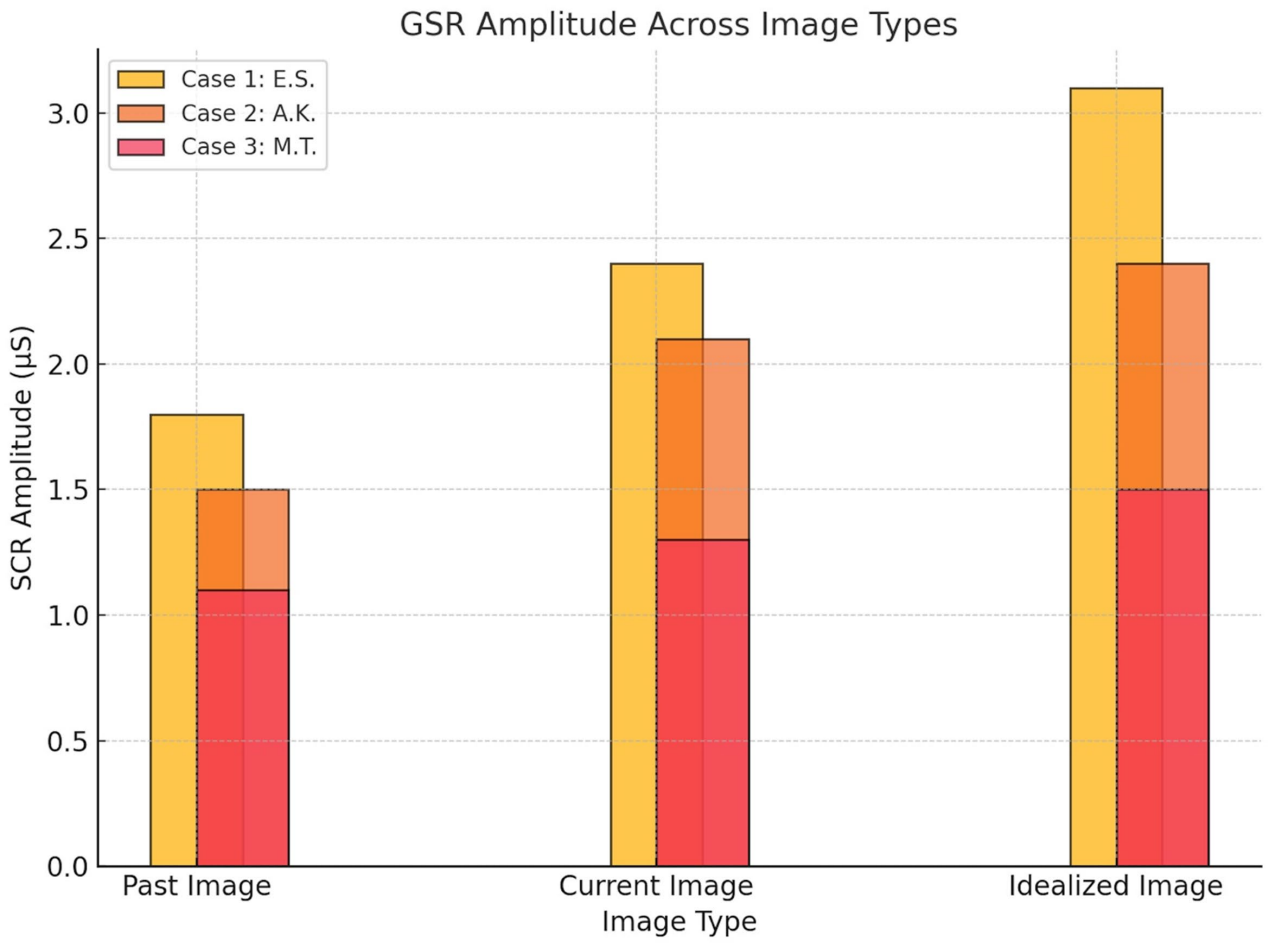
Skin conductance response.

Skin Conductance Response (SCR) amplitudes across image types for each participant. Case 1 (E.S., active MD) showed the strongest autonomic reactivity, particularly to the idealized image. Case 2 (A.K., recovered MD) demonstrated moderate increases, while Case 3 (M.T., control) exhibited the lowest and most stable responses across all conditions (Figure 3).

### 3.3 Case 3: M.T.

Baseline EEG data revealed low beta wave activity across frontal (7.8 μV^2^, SD = 1.9), parietal (7.4 μV^2^, SD = 1.8), and occipital (8.1 μV^2^, SD = 2.0) regions during the neutral state, with minimal variability. During image exposure, beta wave activity remained stable across image types (Frontal: 9.4 μV^2^, SD = 2.5; Parietal: 8.8 μV^2^, SD = 2.1; Occipital: 10.1 μV^2^, SD = 2.3), and statistical comparisons revealed no significant differences between past, current, and idealized images (*p* > 0.05). Baseline GSR showed consistently low arousal levels (SCR Amplitude: 0.5 μS, SD = 0.2), with minimal changes during image exposure (1.3 μS, SD = 0.4; *p* > 0.05). Eye-tracking baseline indicated evenly distributed gaze patterns and short fixation durations (Mean Fixation Duration: 320 ms, Number of Fixations: 6). During image exposure, eye-tracking data continued to show evenly distributed fixations without specific biases (Fixation Duration: 750 ms, Number of Fixations: 12).

Psychological assessments revealed no signs of muscle dysmorphia (MDDI: 0/60), minimal body image distress (BIDQ: 1.8/7), low anxiety (STAI-State: 28/80; STAI-Trait: 30/80), and high self-esteem (RSES: 26/30). No significant correlations emerged between psychological scores and physiological markers (*p* > 0.05).

VAS scores indicated low emotional distress across all images, ranging from 1/10 for the current image to 3/10 for the idealized image. During semi-structured interviews, M.T. described a neutral emotional response to the images, emphasizing his focus on health and overall well-being rather than striving for an idealized physique.

As shown in Table 1, the psychological test results align with the participants’ clinical designations. E.S. presented the most severe profile across all domains, with extreme symptom scores and high emotional reactivity. A.K. exhibited residual vulnerability, reflected in moderate distress and anxiety indicators, while M.T. showed minimal psychological symptoms and high self-esteem. These gradations mirror the physiological and behavioral data patterns described in each case, supporting the internal consistency of the multimodal findings.

**Table 1:** Psychological assessment and VAS ratings summary.

Psychological test/VAS rating	Case 1: E.S. (Current MD and BDD)	Case 2: A.K. (Post-Recovery)	Case 3: M.T. (Control)
MDDI (Muscle Dysmorphic Disorder Inventory)	51/60 (Severe)	18/60 (Mild)	0/60 (None)
BIDQ (Body Image Disturbance Questionnaire)	6.2/7 (High distress)	3.8/7 (Moderate distress)	1.8/7 (Minimal distress)
STAI-State (State Anxiety)	52/80 (Elevated)	40/80 (Moderate)	28/80 (Low)
STAI-Trait (Trait Anxiety)	58/80 (High)	43/80 (Moderate)	30/80 (Low)
RSES (Rosenberg Self-Esteem Scale)	10/30 (Very low)	16/30 (Low-average)	26/30 (High)
VAS-Past Body Image	9	5	2
VAS-Current Body Image	7	7	1
VAS-dealized Body Image	10	6	3

### 3.4 Participants perspective

E.S., the participant with active muscle dysmorphia, described the study as eye-opening, highlighting how the physiological data mirrored his internal emotional struggles. Viewing his past image evoked regret and self-criticism, while the idealized image triggered a mix of aspiration and frustration. He admitted feeling “trapped” by his reliance on steroids, fearing their consequences despite recognizing their harm.

A.K., with a history of muscle dysmorphia, reflected on the emotional complexity of the images. His past image stirred mixed feelings of pride and regret, the current image brought frustration over perceived physical decline, and the idealized image evoked fleeting longing. While his emotional reactivity had decreased, he acknowledged lingering cognitive patterns affecting his self-perception and valued the study for validating his experience.

M.T., the participant with no history of muscle dysmorphia, reported a calm and neutral experience. He viewed the images with curiosity rather than distress or fixation, emphasizing that his fitness motivation stems from health and well-being rather than aesthetic goals. The study reinforced his positive relationship with his body and exercise routine.

## 4 Discussion

This study provides a nuanced understanding of the interplay between psychological and physiological responses in individuals with varying degrees of MD and body image concerns. Baseline measurements provided a neutral reference point, strengthening confidence in the observed stimulus-driven changes in EEG, GSR, and eye-tracking responses across participants. The integration of psychological scales, EEG, GSR, and eye-tracking data offers a multimodal perspective on how cognitive and emotional processes manifest in both subjective experiences and measurable physiological markers. While previous research on BDD has extensively explored physiological responses (Kapsali et al., 2020; Beilharz et al., 2020; Jefferies et al., 2012), this study represents one of the first to apply EEG, GSR, and eye-tracking technologies specifically to individuals with MD, highlighting both shared and distinct patterns between these related disorders. Recent research has further emphasized the distinct nature of male-specific appearance concerns, highlighting the central role of body weight dissatisfaction in muscle dysmorphia and its overlap with eating-related psychopathology (Dal Brun et al., 2024). These findings underscore that male body image disturbance is not merely an extension of traditional BDD or eating disorder models, but rather a domain requiring its own conceptual and diagnostic frameworks—particularly given the salience of muscularity, leanness, and physical control in male populations.

Beyond its novelty, the study’s strength lies in its multimodal triangulation approach, which enhances interpretive depth and internal validity. Triangulation—by integrating subjective self-report data with objective neurophysiological (EEG), autonomic (GSR), and attentional (eye-tracking) indicators—allowed us to identify converging patterns of cognitive-affective disturbance across methods. For instance, participants with high MD symptomatology not only self-reported severe body dissatisfaction and anxiety but also demonstrated elevated beta wave activity in frontal regions, heightened skin conductance reactivity, and prolonged fixations on muscular body regions during image exposure. The convergence of these measures supports the internal coherence of the findings, reducing the likelihood that the observed patterns stem from measurement artifact, demand characteristics, or transient mood states. In psychopathologies such as MD—where insight is often limited and self-report may be affected by social desirability or denial—triangulated data are critical for uncovering implicit emotional and cognitive processes. This layered evidence offers a more reliable and ecologically valid understanding of the psychophysiological mechanisms underpinning MD and sets the stage for more scalable future investigations using this combined measurement framework.

In Case 1 (E.S.), elevated beta wave activity in frontal and occipital regions, coupled with heightened skin conductance responses and prolonged gaze fixation on muscular areas, revealed significant cognitive and emotional distress. These findings align with severe muscle dysmorphia symptoms (MDDI: 51/60) and elevated anxiety scores (STAI-State: 52/80, STAI-Trait: 58/80). Similar patterns have been observed in EEG studies of BDD (Giannopoulos et al., 2021a), where heightened beta activity has been linked to obsessive thought patterns, hyper-vigilance, and emotional dysregulation (Tortella-Feliu et al., 2014; Fusina et al., 2022; Giannopoulos et al., 2021b). However, in MD, the specific fixation on muscular areas, as revealed through eye-tracking data, underscores a more narrowly focused preoccupation compared to the broader physical concerns seen in BDD.

In contrast, Case 2 (A.K.) demonstrated moderate beta wave activity, reflecting residual emotional arousal despite the cessation of steroid use and bodybuilding. While anxiety and body image distress scores (STAI-State: 40/80, BIDQ: 3.8/7) were lower than in Case 1, physiological markers revealed continued sensitivity to body image-related stimuli, particularly in the current image condition. Studies on recovered individuals with BDD (Pagano et al., 2007; Phillips et al., 2006; de la Cruz et al., 2021; Olave et al., 2021) have reported similar lingering cognitive and emotional vulnerabilities, even after symptomatic improvement. This parallel suggests that the residual psychological and physiological traces of MD persist beyond behavioral change, emphasizing the need for sustained psychological support even after symptom remission.

Case 3 (M.T.) presented balanced beta wave activity, stable GSR levels, and evenly distributed eye-tracking patterns, consistent with low psychological distress (MDDI: 0/60, STAI-State: 28/80) and high self-esteem (RSES: 26/30). His physiological responses indicate a neutral emotional state and a healthy psychological relationship with body image, emphasizing the importance of stable self-perception as a protective factor against MD. In contrast to findings in BDD studies (Szentágotai-Tătar et al., 2020; Dietel et al., 2018), where even non-clinical controls often show heightened emotional reactivity when exposed to body-related stimuli, M.T.’s responses suggest a more detached and emotionally regulated engagement with body images.

The study highlights key associations between psychological distress and physiological reactivity, revealing patterns that may serve as early diagnostic markers for MD. Elevated beta wave activity in frontal and occipital regions, increased SCR peaks, and hyper-focused visual attention on muscular areas emerged as consistent indicators of cognitive and emotional dysregulation. Similar markers have been reported in BDD research (Giannopoulos et al., 2022; Ritter et al., 2020), yet MD appears to exhibit a more targeted cognitive and emotional focus on muscularity rather than generalized physical appearance concerns. These findings suggest that a multimodal assessment framework may offer potential utility in characterizing MD-related profiles, and could serve as a foundation for future studies on diagnostic refinement and monitoring.

From a therapeutic perspective, these preliminary results point to the potential value of integrated intervention strategies targeting cognitive, affective, and physiological processes—though further empirical validation is required. Cognitive-behavioral therapy (CBT) remains a cornerstone for addressing cognitive distortions and emotional preoccupations seen in MD (Cunningham et al., 2017). However, physiological markers such as beta wave activity and skin conductance responses could offer real-time feedback during therapeutic interventions, enhancing self-awareness and emotional regulation strategies. Eye-tracking data, particularly fixation patterns, might also inform exposure-based interventions by identifying and disrupting maladaptive visual attention biases. These exploratory findings raise the possibility that biofeedback techniques could complement CBT protocols, a direction that may be valuable to explore in future controlled intervention studies.

The use of a single exposure per image condition was a deliberate methodological choice rooted in both ethical and empirical considerations. Given the emotionally salient and autobiographical nature of the stimuli, repeated viewings risked eliciting distress, response fatigue, or habituation, particularly among participants with active or residual MD symptoms. This concern is consistent with previous research in affective neuroscience and clinical body image studies, which often employ single-trial designs when using personalized or emotionally charged stimuli. Despite the limited number of trials, continuous multimodal recording—coupled with individualized baseline correction—enabled the detection of meaningful within-subject physiological and attentional shifts. Future studies employing neutral or standardized images may benefit from multiple trials per condition to enhance signal averaging and reliability, particularly in larger samples or experimental protocols involving less sensitive populations.

While the limited number of cases restricts statistical generalization, the present findings should be interpreted as part of an exploratory, proof-of-concept framework. The consistent physiological and psychological patterns observed across participants—particularly in beta wave reactivity, autonomic arousal, and visual fixation—suggest potential candidate markers for cognitive-affective dysregulation in MD. These observations are not intended as confirmatory evidence but rather as a foundation for generating empirically testable hypotheses in future experimental research. Larger-scale, controlled studies will be required to validate the proposed psychophysiological profiles and to examine their diagnostic and therapeutic implications.

This case series highlights the value of multimodal assessment in uncovering complex interplays between emotional distress, cognitive patterns, and physiological reactivity in MD. As a hypothesis-generating investigation, it underscores the importance of early-stage research in advancing the neurocognitive understanding of underexplored clinical conditions. Future studies may build upon this preliminary framework to examine diagnostic features, explore targeted interventions, and contribute to the development of a more nuanced understanding of the neurocognitive dimensions of muscle dysmorphia.

## 5 Limitations

Despite offering novel insights, this study has several limitations. The most prominent is the limited sample size, which precludes statistical generalization and reduces the power to detect more nuanced effects or subgroup differences. While a case-series design was deliberately chosen to enable in-depth multimodal analysis of a rare and complex phenomenon, the findings should be interpreted as exploratory and hypothesis-generating rather than confirmatory. Additionally, although steps were taken to mitigate sensor noise and inter-individual variability—such as using baseline-corrected data and repeated-measures analysis—physiological signals like EEG and GSR remain inherently sensitive to extraneous factors including hydration, fatigue, and circadian rhythms. In addition, individual differences in baseline autonomic tone or neurocognitive reactivity—as well as state-dependent variability related to mood, attention, or sleep—may influence physiological responses and reduce the generalizability of these markers. Differences in participants’ prior exposure to psychophysiological assessment, emotional expressivity, and cognitive reflection may also influence outcomes in ways not fully controllable within a small sample. The subjective nature of personalized image stimuli, while ecologically valid, introduces variability in stimulus salience that may impact comparability across individuals. Finally, although qualitative interviews enriched the data interpretation, thematic saturation was not pursued due to the limited number of participants. Future studies employing larger samples, standardized stimuli, and longitudinal tracking are needed to validate these initial findings and refine multimodal indicators of MD.

## Data Availability

The original contributions presented in the study are included in the article/Supplementary material, further inquiries can be directed to the corresponding author.

## References

[ref1] Beilharz F. Phillipou A. Castle D. J. Rossell S. L. (2020). Saccadic eye movements in body dysmorphic disorder. J. Obsessive-Compuls. Relat. Disord. 25:100526. doi: 10.1016/j.jocrd.2020.100526

[ref2] Bonnecaze A. K. O’Connor T. Burns C. A. (2021). Harm reduction in male patients actively using anabolic androgenic steroids (AAS) and performance-enhancing drugs (PEDs): a review. J. Gen. Intern. Med. 36, 2055–2064. doi: 10.1007/s11606-021-06751-3, PMID: 33948794 PMC8298654

[ref3] Cuadrado J. Laulan P. Sentenac C. Legigan C. Michel G. (2024). “Bigger, stronger, sicker”, integrative psychological assessment for muscle dysmorphia: case studies of two young women bodybuilders. Psychiatry Res. Case Rep. 3:100212. doi: 10.1016/j.psycr.2024.100212

[ref4] Cunningham M. L. Griffiths S. Mitchison D. Mond J. M. Castle D. Murray S. B. (2017). Muscle Dysmorphia: an overview of clinical features and treatment options. J. Cogn. Psychother. 31, 255–271. doi: 10.1891/0889-8391.31.4.255, PMID: 32755900

[ref5] Dal Brun D. Pescarini E. Calonaci S. Bonello E. Meneguzzo P. (2024). Body evaluation in men: the role of body weight dissatisfaction in appearance evaluation, eating, and muscle dysmorphia psychopathology. J. Eat. Disord. 12:65. doi: 10.1186/s40337-024-01025-9, PMID: 38773673 PMC11110325

[ref6] de la Cruz L. F. Enander J. Rück C. Wilhelm S. Phillips K. A. Steketee G.. (2021). Empirically defining treatment response and remission in body dysmorphic disorder. Psychol. Med. 51, 83–89. doi: 10.1017/S0033291719003003, PMID: 31662124 PMC7190405

[ref7] Devrim A. Bilgic P. (2019). Validity and reliability study of Turkish version of “muscle dysmorphic disorder inventory” and “bodybuilder image grid” scales. Curr. Nutr. Food Sci. 15, 517–524. doi: 10.2174/1573401314666181012113904

[ref8] Dietel F. A. Möbius M. Steinbach L. Dusend C. Wilhelm S. Buhlmann U. (2018). Effects of induced appearance-related interpretation bias: a test of the cognitive-behavioral model of body dysmorphic disorder. J. Behav. Ther. Exp. Psychiatry 61, 180–187. doi: 10.1016/j.jbtep.2018.07.003, PMID: 30118967

[ref9] Döner S. Efe Y. S. Elmalı F. (2024). Turkish adaptation of the state–trait anxiety inventory short version (STAIS-5, STAIT-5). Int. J. Nurs. Pract. 30:e13304. doi: 10.1111/ijn.13304, PMID: 39323115 PMC11608918

[ref10] Fusina F. Marino M. Spironelli C. Angrilli A. (2022). Ventral attention network correlates with high traits of emotion dysregulation in community women—a resting-state EEG study. Front. Hum. Neurosci. 16:895034. doi: 10.3389/fnhum.2022.895034, PMID: 35721362 PMC9205637

[ref11] Giannopoulos A. E. Spantideas S. T. Capsalis C. Papageorgiou P. Kapsalis N. Kontoangelos K.. (2021a). Instantaneous radiated power of brain activity: application to prepulse inhibition and facilitation for body dysmorphic disorder. Biomed. Eng. Online 20, 108–121. doi: 10.1186/s12938-021-00946-9, PMID: 34689781 PMC8543766

[ref12] Giannopoulos A. E. Zioga I. Kontoangelos K. Papageorgiou P. Kapsali F. Capsalis C. N.. (2022). Deciding on optical illusions: reduced alpha power in body dysmorphic disorder. Brain Sci. 12:293. doi: 10.3390/brainsci12020293, PMID: 35204056 PMC8870663

[ref13] Giannopoulos A. E. Zioga I. Papageorgiou P. C. Kapsali F. Spantideas S. T. Kapsalis N. C.. (2021b). Early auditory-evoked potentials in body dysmorphic disorder: an ERP/sLORETA study. Psychiatry Res. 299:113865. doi: 10.1016/j.psychres.2021.113865, PMID: 33735739

[ref14] Gnambs T. Scharl A. Schroeders U. (2018). The structure of the Rosenberg self-esteem scale. Z. Psychol. 226:317. doi: 10.1027/2151-2604/a000317

[ref15] Greenway C. W. Price C. (2020). Muscle dysmorphia and self-esteem in former and current users of anabolic-androgenic steroids. Perform. Enhan. Health 7:100154. doi: 10.1016/j.peh.2019.100154

[ref16] Jefferies K. Laws K. R. Fineberg N. A. (2012). Superior face recognition in body dysmorphic disorder. J. Obsessive-Compuls. Related Disord. 1, 175–179. doi: 10.1016/j.jocrd.2012.03.002

[ref17] Kapsali F. Zioga I. Papageorgiou P. Smyrnis N. Chrousos G. P. Papageorgiou C. (2020). Event-related EEG oscillations in body dysmorphic disorder. Eur. J. Clin. Investig. 50:e13208. doi: 10.1111/eci.13208, PMID: 31995842

[ref18] Kuzu D. Berk H. Ö. S. Şimşek Ö. F. (2020). Reliability and validity of the Turkish version of the body image disturbance questionnaire-scoliosis. Spine 45, E1033–E1038. doi: 10.1097/BRS.0000000000003477, PMID: 32706567

[ref19] Leaf C. Turner R. Wasserman C. Paulson R. Kopooshian N. Lynch G.. (2023). Psycho-neuro-biological correlates of beta activity. Neuroregulation 10:11. doi: 10.15540/nr.10.1.11

[ref20] Lukacz E. S. Lawrence J. M. Burchette R. J. Luber K. M. Nager C. W. Buckwalter J. G. (2004). The use of visual analog scale in urogynecologic research: a psychometric evaluation. Am. J. Obstet. Gynecol. 191, 165–170. doi: 10.1016/j.ajog.2004.04.047, PMID: 15295359

[ref21] Magaldi D. Berler M. (2020). “Semi-structured interviews” in Encyclopedia of personality and individual differences, eds. Zeigler-Hill, V., and Shackelford, T. K. (Cham: Springer).

[ref22] Martenstyn J. A. Aouad P. Touyz S. Maguire S. (2022a). Treatment of compulsive exercise in eating disorders and muscle dysmorphia: a systematic review and meta-analysis. Clin. Psychol. Sci. Pract. 29, 143–161. doi: 10.1037/cps0000064, PMID: 40165438

[ref23] Martenstyn J. A. Maguire S. Griffiths S. (2022b). A qualitative investigation of the phenomenology of muscle dysmorphia: part 1. Body Image 43, 486–503. doi: 10.1016/j.bodyim.2022.10.009, PMID: 36356368

[ref24] Murray S. B. Rieger E. Touyz S. W. De la Garza García L. Y. (2010). Muscle dysmorphia and the DSM-V conundrum: where does it belong? A review paper. Int. J. Eat. Disord. 43, 483–491. doi: 10.1002/eat.20828, PMID: 20862769

[ref25] Olave L. Estévez A. Momeñe J. Muñoz-Navarro R. Gómez-Romero M. J. Boticario M. J.. (2021). Exercise addiction and muscle dysmorphia: the role of emotional dependence and attachment. Front. Psychol. 12:681808. doi: 10.3389/fpsyg.2021.681808, PMID: 34220650 PMC8250146

[ref26] Pagano M. E. Phillips K. A. Stout R. L. Menard W. Piliavin J. A. (2007). Impact of helping behaviors on the course of substance-use disorders in individuals with body dysmorphic disorder. J. Stud. Alcohol Drugs 68, 291–295. doi: 10.15288/jsad.2007.68.291, PMID: 17286348 PMC2213452

[ref27] Perera M. P. N. Mallawaarachchi S. Bailey N. W. Murphy O. W. Fitzgerald P. B. (2023). Obsessive-compulsive disorder (OCD) is associated with increased electroencephalographic (EEG) delta and theta oscillatory power but reduced delta connectivity. J. Psychiatr. Res. 163, 310–317. doi: 10.1016/j.jpsychires.2023.05.026, PMID: 37245318

[ref28] Phillips K. A. Pagano M. E. Menard W. Stout R. L. (2006). A 12-month follow-up study of the course of body dysmorphic disorder. Am. J. Psychiatry 163, 907–912. doi: 10.1176/ajp.2006.163.5.907, PMID: 16648334 PMC1613833

[ref29] Prnjak K. Jukic I. Mitchison D. Griffiths S. Hay P. (2022). Body image as a multidimensional concept: a systematic review of body image facets in eating disorders and muscle dysmorphia. Body Image 42, 347–360. doi: 10.1016/j.bodyim.2022.07.006, PMID: 35926364

[ref30] Riley D. S. Barber M. S. Kienle G. S. Aronson J. K. von Schoen-Angerer T. Tugwell P.. (2017). Care guidelines for case reports: explanation and elaboration document. J. Clin. Epidemiol. 89, 218–235. doi: 10.1016/j.jclinepi.2017.04.026, PMID: 28529185

[ref31] Ritter V. Kaufmann J. M. Krahmer F. Wiese H. Stangier U. Schweinberger S. R. (2020). Neural correlates of own-and other-face perception in body dysmorphic disorder. Front. Psychol. 11:302. doi: 10.3389/fpsyt.2020.00302, PMID: 32395110 PMC7196670

[ref32] Rück C. Mataix-Cols D. Feusner J. D. Shavitt R. G. Veale D. Krebs G.. (2024). Body dysmorphic disorder. Nat. Rev. Dis. Prim. 10, 92–15. doi: 10.1038/s41572-024-00577-z, PMID: 39639018 PMC12032537

[ref33] Shehu H. A. Oxner M. Browne W. N. Eisenbarth H. (2023). Prediction of moment-by-moment heart rate and skin conductance changes in the context of varying emotional arousal. Psychophysiology 60:e14303. doi: 10.1111/psyp.14303, PMID: 37052214

[ref34] Sreshta N. Pope H. G. Hudson J. I. Kanayama G. (2017). “Muscle dysmorphia” in Body dysmorphic disorder: advances in research and clinical practice, 81.

[ref35] Szentágotai-Tătar A. Nechita D. M. Miu A. C. (2020). Shame in anxiety and obsessive-compulsive disorders. Curr. Psychiatry Rep. 22, 1–9. doi: 10.1007/s11920-020-1142-9 32076847

[ref36] Tortella-Feliu M. Morillas-Romero A. Balle M. Llabrés J. Bornas X. Putman P. (2014). Spontaneous EEG activity and spontaneous emotion regulation. Int. J. Psychophysiol. 94, 365–372. doi: 10.1016/j.ijpsycho.2014.09.003, PMID: 25219892

[ref37] Wong W. W. Rangaprakash D. Diaz-Fong J. P. Rotstein N. M. Hellemann G. S. Feusner J. D. (2022). Neural and behavioral effects of modification of visual attention in body dysmorphic disorder. Transl. Psychiatry 12:325. doi: 10.1038/s41398-022-02099-2, PMID: 35948537 PMC9365821

[ref38] Zaiser C. Laskowski N. M. Müller R. Abdulla K. Sabel L. Ballero C.. (2024). The relationship between anabolic androgenic steroid use and body image, eating behavior, and physical activity by gender: a systematic review. Neurosci. Biobehav. Rev.:105772. doi: 10.1016/j.neubiorev.2024.105772, PMID: 38879097

[ref39] Zheng Y. Zhang L. Shao P. Guo X. (2021). The association of muscle dysmorphia, social physique anxiety, and body checking behavior in male college students with weight exercise. Front. Psychol. 12:726032. doi: 10.3389/fpsyg.2021.726032, PMID: 34630239 PMC8497756

