# Supplementary material for: Case Report: Physiological and psychological underpinnings of muscle dysmorphia using EEG, GSR, and eye-tracking
Source: Front Psychol. 2025 Jul 21;16:1553997. doi: 10.3389/fpsyg.2025.1553997 (PMC12320501; doi:10.3389/fpsyg.2025.1553997)
Supplement: Supplementary file 1 [file Supplementary_file_1.docx]

**1. Muscle Dysmorphic Disorder Inventory (MDDI)**

**Questionnaire Template**

The MDDI consists of 13 items that measure three subscales: **Drive for Size**, **Appearance Intolerance**, and **Functional Impairment**. Participants respond on a 5-point Likert scale, where:

- **1 = Never**
- **2 = Rarely**
- **3 = Sometimes**
- **4 = Often**
- **5 = Always**

**Example Items:**

1. I feel dissatisfied with my overall body size.
2. I find it difficult to focus on anything other than my appearance.
3. My workout schedule interferes with my social life.

**Scoring Guidelines**

- **Total Score:** Add scores for all 13 items (range: 13–65).
- **Interpretation:**
  - 13–30: Minimal symptoms
  - 31–45: Moderate symptoms
  - 46–65: Severe symptoms

**2. Body Image Disturbance Questionnaire (BIDQ)**

**Questionnaire Template**

The BIDQ is a 7-item scale measuring distress and preoccupation with body image. Participants rate their agreement on a 7-point Likert scale, where:

- **1 = Not at all**
- **7 = Very severely**

**Example Items:**

1. How often do you think about your physical appearance?
2. How much distress do these thoughts cause you?
3. How often do these thoughts interfere with your daily activities?

**Scoring Guidelines**

- **Total Score:** Average the scores of all 7 items (range: 1–7).
- **Interpretation:**
  - 1–2: Minimal distress
  - 3–4: Moderate distress
  - 5–7: Severe distress

**3. State-Trait Anxiety Inventory (STAI)**

**Questionnaire Template**

The STAI consists of two subscales (State and Trait Anxiety), each with 20 items. Participants rate their responses on a 4-point scale:

- **1 = Not at all**
- **2 = Somewhat**
- **3 = Moderately so**
- **4 = Very much so**

**Example Items (State Anxiety):**

1. I feel calm.
2. I am tense.

**Example Items (Trait Anxiety):**

1. I feel secure.
2. I feel inadequate.

**Scoring Guidelines**

- Reverse scoring for positively worded items (e.g., “I feel calm”).
- **Total Score (State or Trait):** Sum all item scores (range: 20–80).
- **Interpretation:**
  - 20–39: Low anxiety
  - 40–59: Moderate anxiety
  - 60–80: High anxiety

**4. Rosenberg Self-Esteem Scale (RSES)**

**Questionnaire Template**

The RSES includes 10 items measuring self-esteem on a 4-point scale:

- **1 = Strongly Disagree**
- **2 = Disagree**
- **3 = Agree**
- **4 = Strongly Agree**

**Example Items:**

1. I feel that I am a person of worth, at least on an equal basis with others.
2. I feel that I have a number of good qualities.

**Scoring Guidelines**

- Reverse scoring for negatively worded items (e.g., “At times, I think I am no good at all”).
- **Total Score:** Sum all item scores (range: 10–40).
- **Interpretation:**
  - 30–40: High self-esteem
  - 20–29: Moderate self-esteem
  - 10–19: Low self-esteem
