# Supplementary material for: Case Report: Physiological and psychological underpinnings of muscle dysmorphia using EEG, GSR, and eye-tracking
Source: Front Psychol. 2025 Jul 21;16:1553997. doi: 10.3389/fpsyg.2025.1553997 (PMC12320501; doi:10.3389/fpsyg.2025.1553997)
Supplement: Supplementary file 2 [file Supplementary_file_2.docx]

**Pre-Processing Pipelines**

**1. EEG Data Cleaning and Processing**

The following steps were applied to ensure the accuracy and reliability of EEG data:

1. **Artifact Rejection:**
   - **Criteria:** Data segments containing muscle movements, eye blinks, or electrode drifts were identified and excluded.
   - **Automated Detection:** Algorithms flagged signal spikes exceeding ±75 µV or showing abrupt changes over <100 ms.
   - **Visual Inspection:** Trained researchers reviewed flagged segments to confirm artifacts before removal.
2. **Filtering:**
   - **Bandpass Filter:** Applied at 0.5–50 Hz to remove very low-frequency drifts and high-frequency noise.
   - **Notch Filter:** Applied at 50 Hz to eliminate powerline noise.
3. **Segmentation:**
   - EEG data were segmented into epochs corresponding to each image viewing period (30 seconds per image) with 5-second buffers before and after the stimulus.
4. **Baseline Correction:**
   - A baseline segment of 5 seconds (neutral gray screen) preceding each image was used to normalize beta wave activity.
5. **Beta Wave Extraction:**
   - Beta band power (13–30 Hz) was calculated for frontal (F3, F4), parietal (P3, P4), and occipital (O1, O2) regions.
   - Spectral analysis was conducted using the Fast Fourier Transform (FFT) to compute power spectral densities.
6. **Quality Control:**
   - Data with more than 20% artifacts across all epochs or channels were excluded from analysis.

**2. GSR Pre-Processing**

1. **Baseline Correction:**
   - A 1-minute baseline segment was recorded before the task to establish a stable reference for skin conductance.
2. **Smoothing:**
   - Raw GSR signals were smoothed using a moving average filter (window size: 200 ms) to reduce noise.
3. **Peak Detection:**
   - **Threshold:** Peaks were defined as conductance changes exceeding 0.05 µS from the baseline.
   - **Latency and Amplitude:** Peaks were timestamped and measured for response latency (time from stimulus onset to peak) and amplitude (maximum conductance change).
4. **Normalization:**
   - SCR amplitudes were normalized by subtracting the participant’s baseline level to account for individual variability.
5. **Artifact Removal:**
   - Sudden signal drops or spikes exceeding physiologically plausible limits (e.g., >10 µS) were flagged and removed.

**3. Eye-Tracking Calibration and Quality Checks**

1. **Calibration Procedure:**
   - A 9-point calibration grid was used, requiring participants to fixate on a moving dot displayed sequentially at predefined screen locations.
   - Calibration was repeated until an average error of ≤0.5° visual angle was achieved across points.
2. **Validation:**
   - Participants viewed a validation grid of 5 random points. Calibration accuracy was confirmed by comparing actual gaze coordinates with target positions.
3. **Fixation and Saccade Detection:**
   - Fixations were defined as gaze points maintained within a 1° visual angle for at least 100 ms.
   - Saccades were identified as rapid eye movements exceeding 30°/s.
4. **Data Quality Checks:**
   - Trials with excessive blinks or loss of tracking (>20% of total viewing time) were excluded.
   - Heatmaps and gaze path plots were visually inspected for anomalies.
5. **AOI Analysis:**
   - Predefined areas of interest (AOIs) on the images (e.g., chest, arms) were mapped. Fixation durations and counts were computed for each AOI to assess participant focus.
