# Supplementary material for: Case Report: Physiological and psychological underpinnings of muscle dysmorphia using EEG, GSR, and eye-tracking
Source: Front Psychol. 2025 Jul 21;16:1553997. doi: 10.3389/fpsyg.2025.1553997 (PMC12320501; doi:10.3389/fpsyg.2025.1553997)
Supplement: Supplementary file 3 [file Supplementary_file_3.docx]

**Experimental Protocol**

**Step-by-Step Procedure**

1. **Participant Arrival and Orientation (10 Minutes)**
   - Upon arrival at the **Üsküdar University Psycho-Physical Laboratory**, participants were welcomed and provided with an overview of the study aims and procedures.
   - Participants reviewed and signed the informed consent form, confirming their understanding of the protocol and their right to withdraw at any time.
2. **Baseline Psychological Assessment (15 Minutes)**
   - Participants completed four psychological scales to assess body image distress, anxiety, and self-esteem:
     - **Muscle Dysmorphic Disorder Inventory (MDDI)**
     - **Body Image Disturbance Questionnaire (BIDQ)**
     - **State-Trait Anxiety Inventory (STAI)**
     - **Rosenberg Self-Esteem Scale (RSES)**
   - Instructions were provided verbally, and participants filled out the scales in a quiet, comfortable room to ensure focus.
3. **Preparation for Physiological Measurements (20 Minutes)**
   - Participants were seated in an ergonomically designed chair approximately 60 cm from a high-resolution 27-inch monitor.
   - **EEG Setup:**
     - A 64-channel EEG system was calibrated following the International 10-20 system.
     - Conductive gel was applied to ensure optimal signal quality, and impedance levels were checked to remain below 5 kΩ for all electrodes.
   - **GSR Setup:**
     - GSR electrodes were attached to the non-dominant hand's index and middle fingers. Calibration was performed to ensure baseline stability for skin conductance.
   - **Eye-Tracking Calibration:**
     - Eye-tracking was calibrated using a 9-point gaze calibration procedure integrated into the OpenSiSEM platform. Participants were instructed to follow a moving dot on the screen to align gaze measurements.
4. **Image Viewing Task (10 Minutes)**
   - Participants viewed three images in randomized order:
     - **Past Body Image:** A photograph from an earlier stage of life.
     - **Current Body Image:** A recent photograph of their physique.
     - **Idealized Body Image:** A standardized fitness model image.
   - Each image was displayed for **30 seconds**, followed by a **10-second neutral gray screen** to reset physiological responses.
   - Participants were instructed to remain still, focus on the images, and avoid closing their eyes during the task.
5. **Post-Exposure Emotional Assessment (10 Minutes)**
   - After each image, participants rated their emotional response on a **Visual Analog Scale (VAS)** ranging from 0 (no distress) to 10 (extreme distress).
   - A semi-structured interview followed the image-viewing task, during which participants elaborated on their feelings and thoughts evoked by the images. Responses were audio-recorded for qualitative analysis.
6. **Debriefing and Follow-Up Support (5 Minutes)**
   - Participants were thanked for their participation and offered an explanation of their physiological and emotional responses.
   - Those reporting significant distress were referred to follow-up psychological support services.

**Calibration and Setup Details**

1. **EEG Calibration**
   - A test signal was generated to ensure all electrodes were functioning correctly.
   - Artifact rejection filters were applied in real-time to exclude muscle movements and eye blinks.
2. **GSR Calibration**
   - A baseline measurement was taken for one minute before the task. Participants were instructed to relax and breathe naturally.
3. **Eye-Tracking Calibration**
   - Gaze accuracy was confirmed using a fixation dot presented at different locations on the screen. Calibration was repeated if the system detected errors.

**Visual Flowchart of the Experimental Procedure**

1. **Arrival and Orientation (10 Minutes)**
   ↓
2. **Baseline Psychological Assessment (15 Minutes)**
   ↓
3. **Setup and Calibration for EEG, GSR, and Eye-Tracking (20 Minutes)**
   ↓
4. **Image Viewing Task (10 Minutes)**
   - Randomized order of images
   - 30 seconds per image
   - 10-second neutral gray screen between images
     ↓
5. **Post-Exposure Emotional Assessment (10 Minutes)**
   - VAS Scoring
   - Semi-Structured Interview
     ↓
6. **Debriefing and Follow-Up (5 Minutes)**
