# Supplementary material for: Case Report: Physiological and psychological underpinnings of muscle dysmorphia using EEG, GSR, and eye-tracking
Source: Front Psychol. 2025 Jul 21;16:1553997. doi: 10.3389/fpsyg.2025.1553997 (PMC12320501; doi:10.3389/fpsyg.2025.1553997)
Supplement: Supplementary file 4 [file Supplementary_file_4.docx]

**Supplementary Statistical Analysis**

**EEG Beta Wave Activity**

The EEG beta wave activity (13–30 Hz) across the frontal, parietal, and occipital regions was analyzed for each image type (past, current, and idealized). Repeated-measures ANOVA was conducted to examine the effect of image type on beta wave activity across the three cases.

- **Case 1 (E.S.):** Significant differences in beta activity were observed across image types (F(2, 59) = 6.89, p = 0.002, η² = 0.19). Post-hoc tests revealed significantly higher beta activity for the idealized image compared to the past image (p = 0.001) and the current image (p = 0.02).
- **Case 2 (A.K.):** A moderate effect of image type was found (F(2, 59) = 5.22, p = 0.009, η² = 0.17), with significantly higher beta activity for the current image compared to the past image (p = 0.03) and no significant difference between the idealized and past images (p = 0.08).
- **Case 3 (M.T.):** No significant differences were observed in beta activity across image types (F(2, 59) = 2.14, p = 0.13, η² = 0.06).

**GSR Amplitude**

Skin conductance response (SCR) amplitudes were analyzed to assess autonomic arousal during the image viewing task. Repeated-measures ANOVA was conducted to compare arousal levels across image types.

- **Case 1 (E.S.):** Significant differences were found in SCR amplitudes across image types (F(2, 59) = 9.21, p < 0.001, η² = 0.24). The idealized image elicited significantly higher SCR amplitudes than the past image (p = 0.001) and the current image (p = 0.003).
- **Case 2 (A.K.):** Moderate differences were observed (F(2, 59) = 4.86, p = 0.01, η² = 0.14). The current image elicited significantly higher SCR amplitudes than the past image (p = 0.02), with no significant difference between the current and idealized images (p = 0.09).
- **Case 3 (M.T.):** No significant differences in SCR amplitudes were observed across image types (F(2, 59) = 1.89, p = 0.17, η² = 0.05).

**Eye-Tracking Metrics**

Fixation duration was analyzed to measure visual attention across the areas of interest (AOIs) for each image type. A mixed-effects model was used to assess the interaction between image type and AOIs.

- **Case 1 (E.S.):** Significant interaction between image type and AOI was found (F(4, 177) = 11.42, p < 0.001). Prolonged fixation durations were observed on the arms and chest in the idealized image compared to the past image (p = 0.002).
- **Case 2 (A.K.):** Moderate interaction was found (F(4, 177) = 8.75, p < 0.001), with greater attention to perceived weaker areas (e.g., arms, abdomen) in the current image compared to the past image (p = 0.03).
- **Case 3 (M.T.):** Minimal interaction between image type and AOI was observed (F(4, 177) = 1.74, p = 0.15), reflecting balanced gaze distribution across all image types.

**Correlations Between Physiological and Psychological Measures**

Pearson’s correlation analysis was conducted to assess relationships between physiological measures (EEG, GSR) and psychological scale scores (MDDI, BIDQ, STAI, RSES).

- **Case 1 (E.S.):**
  - Beta activity for the current image was strongly correlated with MDDI scores (r = 0.72, p < 0.01).
  - SCR amplitude for the idealized image was positively correlated with BIDQ scores (r = 0.65, p < 0.01).
  - Low self-esteem (RSES scores) was negatively correlated with beta activity for the past image (r = -0.63, p < 0.01).
- **Case 2 (A.K.):**
  - SCR amplitude for the current image was moderately correlated with MDDI scores (r = 0.54, p < 0.05).
  - Fixation duration on weaker areas in the current image was positively correlated with BIDQ scores (r = 0.62, p < 0.01).
- **Case 3 (M.T.):**
  - No significant correlations were observed, consistent with the absence of MD symptoms and minimal body image distress.
